# Applications and analytical tools of cell communication based on ligand-receptor interactions at single cell level

**DOI:** 10.1186/s13578-021-00635-z

**Published:** 2021-07-03

**Authors:** Fen Ma, Siwei Zhang, Lianhao Song, Bozhi Wang, Lanlan Wei, Fengmin Zhang

**Affiliations:** 1 Department of Microbiology, Harbin Medical University, Harbin, 150081 China; 2 Wu Lien-Teh Institute, Harbin Medical University, Harbin, 150081 China; 3 Shenzhen Third People‘s Hospital, Second Hospital, Affiliated to Southern University of Science and Technology, Shenzhen, 518112 China

**Keywords:** Cell communication, Ligand-receptor interactions, Single cell RNA sequencing, Target therapy, Tumor microenvironment

## Abstract

**Background:**

Cellular communication is an essential feature of multicellular organisms. Binding of ligands to their homologous receptors, which activate specific cell signaling pathways, is a basic type of cellular communication and intimately linked to many degeneration processes leading to diseases.

**Main body:**

This study reviewed the history of ligand-receptor and presents the databases which store ligand-receptor pairs. The recently applications and research tools of ligand-receptor interactions for cell communication at single cell level by using single cell RNA sequencing have been sorted out.

**Conclusion:**

The summary of the advantages and disadvantages of analysis tools will greatly help researchers analyze cell communication at the single cell level. Learning cell communication based on ligand-receptor interactions by single cell RNA sequencing gives way to developing new target drugs and personalizing treatment.

## Background

Every cell in multicellular organisms lives in a variety of signaling environments, and its biological behavior is regulated by extracellular signaling molecules. Cell communication is a basic feature of multicellular organisms [1]. The dynamic communicating network formed through communication and cooperation between cells plays crucial roles in numerous biological processes [2, 3]. By specifically recognizing and binding with signal molecules, receptors converting these molecules into intracellular signals and perform specific physiological functions. Complex cellular reactions begin with the binding of ligands to their homologous receptors, which activate specific cell signaling pathways [4]. Therefore, the analysis of ligand-receptor pairs interactions are the basis for understanding cell behavior and responses to neighboring cells.

Recently, Single-cell RNA sequencing (scRNA-seq) has led to breakthroughs in scientific research. scRNA-seq conducted analysis on cellular basis, which makes it feasible to investigate undiscovered cellular commutations. There are some progresses that have been learning intercellular communication through scRNA-seq, which continues to advance at such a rapid pace that even recent reviews [3, 5, 6]. Many research has focued on ligand-receptor interaction-based strategy to construct cellular communication network, rather than the physically vicinal structure-based strategy [3]. Ligand-receptor interactions are effective way to learn cellular communication at single cell level.

Here, we reviewed the recently research progress of ligand-receptor interaction for intercellular communication through scRNA-seq under multiple conditions, and compared the analysis tools for ligand-receptor interactions on single-cell level. These will help to better understand the crucial role of ligand-receptor interactions in cell communication research.

## Ligand-receptor pairs in disease

In 1971, the receptor was first reported to play an important role in cancer with estrogen receptor expressed dysregulated in breast cancer patients [7]. Later, Obesity and hyperinsulinemia were later found to be caused by insulin receptor deficiency in mice [8]. The relationship between ligand-receptor and disease has been discovered gradually. Ligands and receptors are involved in disease development mainly in two ways: structural or genetic alterations and the expression of receptor/ligand change. Familial Hypercholesterolemia (FH), which is caused by low density lipoprotein receptor (LDLR) deficiency [9], and diabetes mellitus which is a consequence of abnormal insulin receptor.

Immune checkpoint is a common and widely studied receptor ligand in tumor research. They are ligand-receptor pairs that inhibit the interaction of the immune response. Cytotoxic T lymphocyte-associated antigen-4 (CTLA-4) was the first immune checkpoint receptor identified. Studies have shown that CTLA-4 is closely related to tumor progression and treatment, and blocking the inhibitory effect of CTLA-4 can enhance the effective immune response against tumor cells [10]. Subsequently, researchers found that various tumor cells can inhibit the function of T cells by working on the immune checkpoint programmed death 1 (PD-1), which allows the tumor cells to escape from immune surveillance [11, 12]. Moreover, the expression of immune checkpoints, such as CD137 (4-1BB), inducible co-stimulator (ICOS), T cell immunoglobulin and mucin domain 3 (TIM-3) changed in the tumor microenvironment could affect tumor progression [13]. The understanding of ligand-receptor interaction is the foundation for current studies of intercellular communication. It gives researchers a deeper insight into the processes of cellular biological activity and disease progression.

With the increasing discovery on receptors and ligands and their interactions, compilation by sorting and summarizing relevant information into ligand-receptor databases has been done continuously to facilitate research (Table 1). Although these databases comprehensively organize the available information on ligand-receptor interaction, there are still undiscovered receptors, ligands and their relationships. Therefore, after analyzing the existing ligand-receptor complexes, researchers developed simulation analysis software for the prediction of ligand-receptor interactions, for example, DOCK [14], Autodock [15, 16], AutoDock Vina, iGEMDOCK, and RosettaDock [17]. Numerous inductive databases and simulation tools help researchers to better study ligand-receptor complexes and their interactions, which in turn contributes to drug development and disease treatment.

**Table 1:** The databases of ligand-receptor pairs

Database	Ligand-receptor complexs	Level	Species	Pairs number	Verified	Address	Author
The database of interacting proteins (DIP) [18, 19]	Yes	Protein	*Homo sapiens*, * Mus musculus*,* Rattus norvegicus*, *Saccharomyces cerevisiae*,* Drosophila melanogaster*,* Escherichia coli*,* Caenorhabditis elegans*,* Helicobacter pylori*, *Bos taurus, Arabidopsis thaliana*	81,923	Yes	http://dip.doe-mbi.ucla.edu	Lukasz Salwinski et al.
Database of ligand-receptor partners (DLRP) [20]	No	Protein	NA	NA	Yes	NA	Graeber et al.
Human plasma membrane receptome (HPMR) database [21]	No	Protein	*Homo sapiens*	NA	Yes	http://Receptome.Stanford.edu	Izhar Ben-Shlomo et al.
The Online Predicted Human Interaction Database (OPHID) [22]	Yes	Protein	*Homo sapiens*,*Saccharomyces cerevisiae*,* Caenorhabditis elegans*,* Drosophila melanogaster *and* Mus musculus*	23,889	Part	http: //ophid.utoronto.ca	Kevin R Brown et al.
Mother Of All Databases (Binding MOAD) [23]	Yes	Protein	NA	38,702	Yes	http://www.BindingMOAD.org	Liegi Hu et al.
Unified Human Interactome database (UniHI) [24]	No	Protein	*Homo sapiens*	150,000	Part	http://www.mdc-berlin.de/unihi	Gautam Chaurasia et al.
Interolog interaction database (I2D) [25]	No	Protein	*Homo sapiens*,*Saccharomyces cerevisiae*,* Caenorhabditis elegans*,* Rattus norvegicus*,* Drosophila melanogaster *and* Mus musculus*	1,279,157	Part	http://ophid.utoronto.ca/i2d/	Kevin R Brown et al.
GPCR-Ligand Database (GLIDA) [26]	Yes	Protein	NA	39,140	Yes	http://pharminfo.pharm.kyoto-u.ac.jp/services/glida/	Yasushi Okuno et al.
ConsensusPathDB [27–29]	No	Protein and gene	*Homo sapiens*	215 541	Part	http://ConsensusPathDB.org	Atanas Kamburov et al.
The International Union of Basic and Clinical Pharmacology database (IUPHAR-DB) [30, 31]	Yes	Protein and gene	*Homo sapiens*	48,902	Part	http://www.iuphar-db.org	Sharman et al.
The molecular interaction database (MINT) [32]	No	Protein	NA	235,000	Part	http://mint.bio.uniroma2.it/mint/	Luana Licata et al.
InnateDB [33]	No	Protein and gene	*Homo sapiens*,* Mus musculus* and* Bos taurus*	18,780	Yes	http://www.innatedb.com	Karin Breuer et al.
The STRING database [34, 35]	No	Protein	5090 organisms	3,123,056,667	Part	http://string-db.org/	Izhar Ben-Shlomo et al.
The TissueNet database [36]	No	Protein	*Homo sapiens*	NA	Part	http://netbio.bgu.ac.il/tissuenet/	Ruth Barshir et al.
The Transformer database [37]	Yes	Protein	NA	NA	Yes	http://bioinformatics.charite.de/transformer	Michael F Hoffmann et al.
IntAct [38]	No	Protein and gene	Multiple organisms	NA	Part	http://www.ebi.ac.uk/intact	Sandra Orchard
The extracellular matrix interaction database (MatrixDB) [39]	No	Protein	Multiple organisms	106,543	Yes	http://matrixdb.ibcp.fr	G Launay et al.
A draft network of ligand-receptor-mediated [40]	No	Protein and gene	*Homo sapiens*	2950	Part	http://fantom.gsc.riken.jp/5/	Ramilowski et al.
The Research Collaboratory for Structural Bioinformatics Protein Data Bank (RCSB PDB) [41]	Yes	Protein	Multiple organisms	NA	Part	http://rcsb.org	Peter W Rose et al.
The DifferentialNet database [42]	No	Protein	*Homo sapiens*	NA	No	http://netbio.bgu.ac.il/diffnet/	Omer Basha et al.
Protein–Protein Interaction Sitesbase (PPInS) [43]	Yes	Protein	NA	32,468	Part	http://www.cup.edu.in:99/ppins/home.php	Vicky Kumar et al.
UniLectin3D [44]	Yes	Protein	NA	NA	Part	https://www.unilectin.eu/unilectin3D	François Bonnardel et al.
Ligand/Receptor Interaction Database (LRdb) [45]	No	Protein and gene	*Homo sapiens*	3085	Yes	https://github.com/SCA-IRCM/LRdb	Simon Cabello-Aguilar et al.
CellPhoneDB [46]	Yes	Protein and gene	*Homo sapiens*	1396	Part	https://www.cellphonedb.org/	Mirjana Efremova et al.
CellTalk Database (CellTalkDB) [47]	No	Protein	*Homo sapiens*, Mouse	5431	Yes	http://tcm.zju.edu.cn/celltalkdb	Xin Shao et al.

Ligand-receptor complexes: if the structures of ligand-receptor comlpexes were considered by the databases

Verified: if the pairs in these databases have been verified

*NA* not available

## scRNA sequencing

With the development of research, the researchers found that different types of cells in the same sample (such as tissue, blood) have different function. Studies have shown that research methods which measure characteristics only on population level may average or dilute important differences between cells. Due to the lack of synchronization among cells, stochastic events of protein production are difficult to observe directly with measurements on large ensembles of cells [48, 49]. Sequencing technology has developed from the first generation to the third generation based on nanopore and single molecule real-time sequencing [50, 51], which only improves the depth, accuracy and throughput of sequencing. However, none of them can re-establish cellular barriers and analyze gene expression at single cell level. Such shortcoming poses a problem for the study of tissue, blood, and other experimental samples consisting of mix multiple cells types. To address such problem, single cell sequencing have been developed to perform high-throughput sequencing of the genome, transcriptome, epigenome, etc. at single cell level [52].

**Fig. 1:**
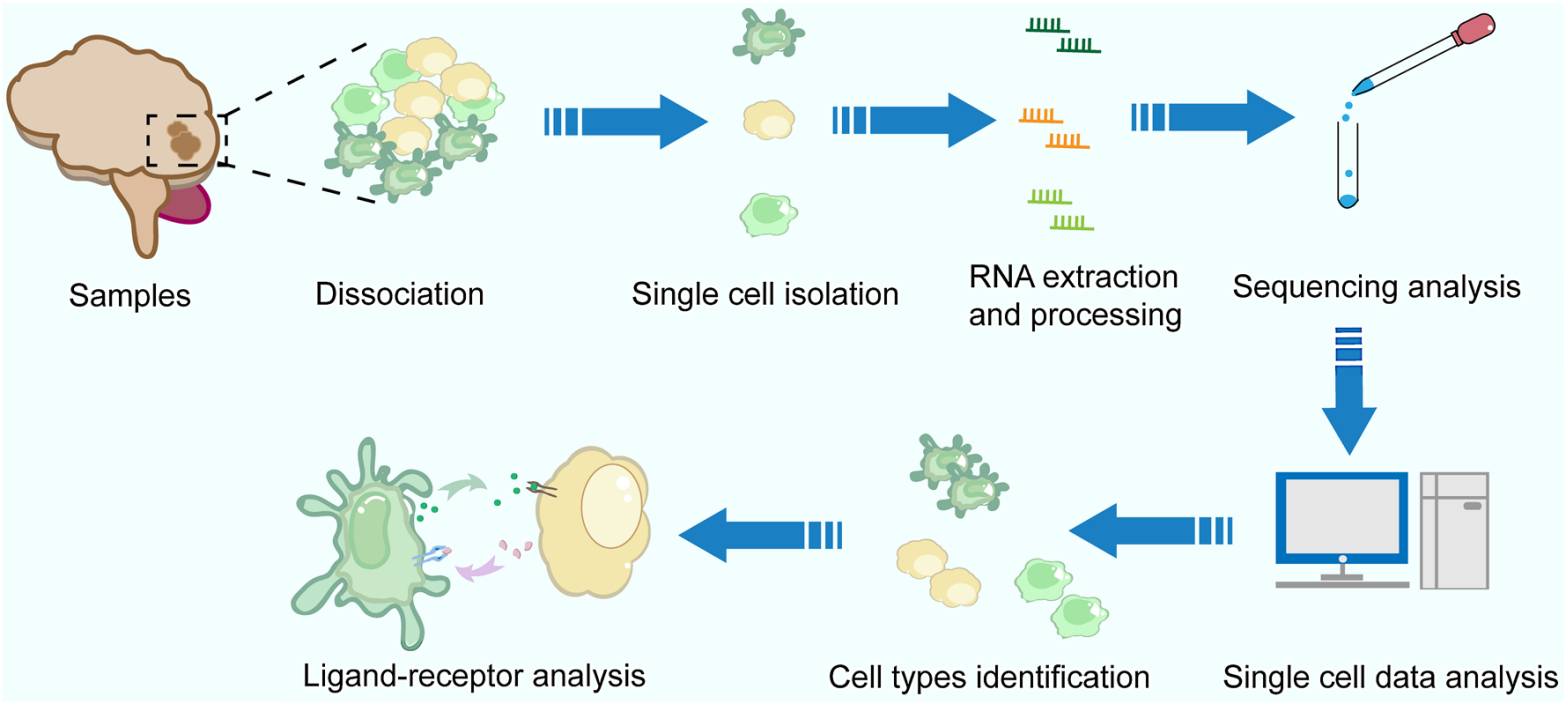
General procedures of ligand-receptor studies using scRNA-seq techniques. multicellular samples were isolated and captured individual cells. All RNA from each cell was reverse transcribed, amplified and sequenced to obtain transcriptome data for each cell in the sample. Cell types were identified. Then, ligand-receptor interaction could analysis by multiple analysis tools

Among them, scRNA-seq was first reported in 2009 [53] by separating single oocytes in Eppendorf tubes containing a lysis buffer [54]. scRNA-seq have enabled the simultaneous classification of thousands of cells in a single assay based on transcriptome profiling [53], which means several novel or rare cell-types that have opportunities to be discovered. The Human Cell Atlas [55] and NIH Brain Initiative projects [56] intend to sequence all cell types present in the human body and brain, respectively. Single-cell transcriptomic atlases provide unprecedented resolution to reveal complex cellular events and deepen our understanding of biological systems [57]. More importantly, the advances of scRNA-seq provide the possibilities to investigate undiscovered cellular commutations (Fig. 1).

## Applications of scRNA sequencing in receptor-ligand analysis

**Fig. 2:**
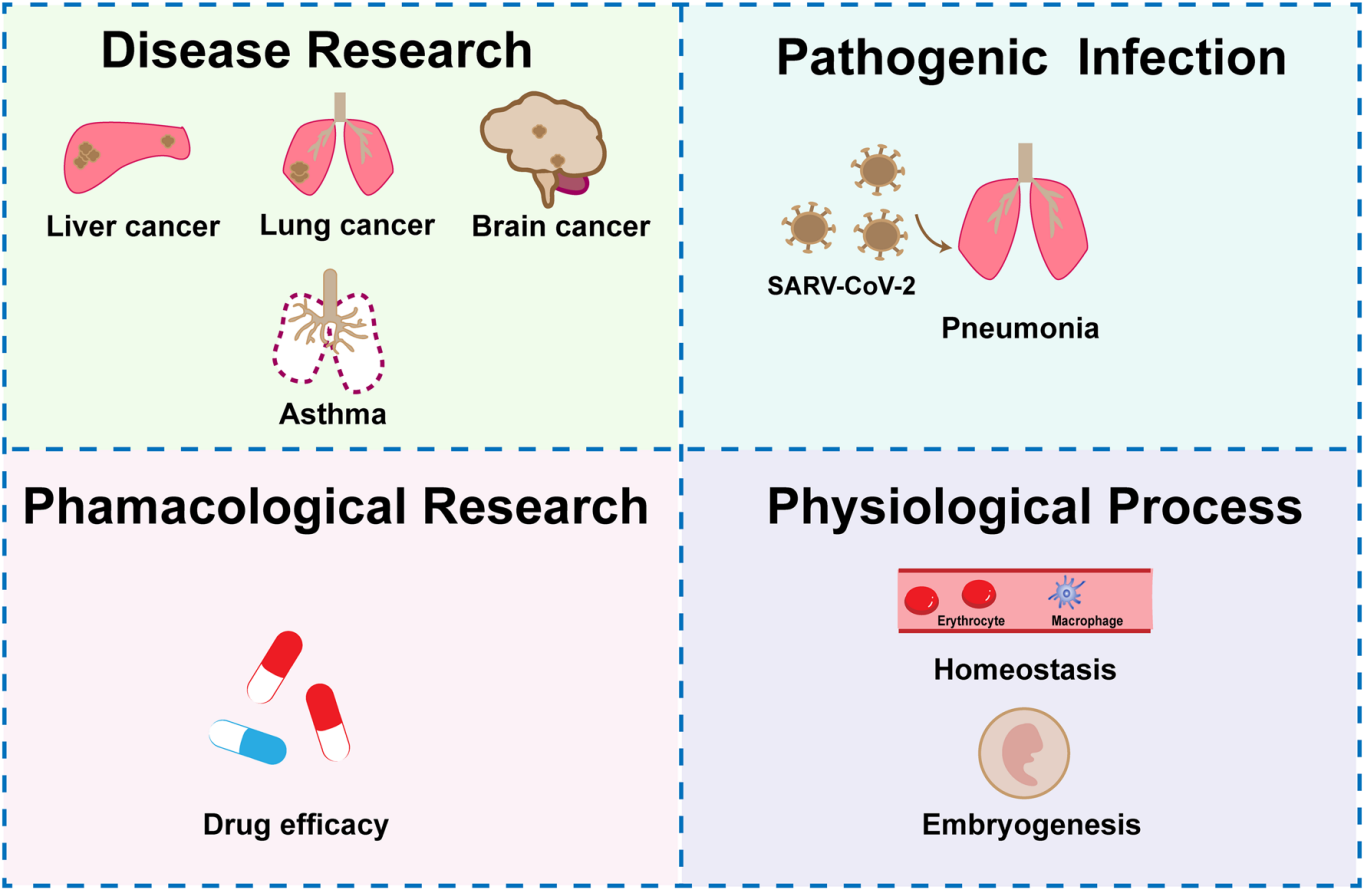
Current applications of scRNA sequencing in ligand-receptor analysis. The analysis of ligand-receptor interactions using scRNA sequencing can be applicate to elucidate in-depth mechanisms underlying disease research, pathogenic infection, physiological process, pharmacological research

The scRNA sequencing has been applied in various research fields to learn the important roles of ligand-receptor interactions in such cellular communications (Fig. 2).

### Disease research

Currently, malignant tumors are one of the main threats to human life. Zemin Zhang et al. found that dendritic cells possess the highest number of ligands, while T cells possess the highest number of receptors, by using scRNA-seq technology: 10 × genomics and Smart-seq2, to analyze cell communications in liver cancer tissues. The main role of dendritic cells in tumor immunity is tumor antigens presentation, migration to lymph nodes, activation of T cells, etc. [58]. After exploring the intercellular communications between cancer stem-like cells (CSCs) and macrophages in glioma through 10 × genomics, Dongsheng Yuan et al. identified 66 ligand-receptor pairs, some of which could significantly affect prognostic outcomes [59]. Using 10 × genomics, Zhencong Chen et al.. analyzed infiltrating tumor cells, epithelial cells, and T cells which are identified in the lung adenocarcinoma (LUAD) tumor microenvironment and sequenced by Smart-seq2, built a valid prognostic machine-learning model based on ligand-receptor interactions for predicting the prognosis of LUAD patients [60]. scRNA-seq is used to analyze the communication between tumor cells and immune cells in tumor microenvironment, helping researchers to better analyze tumor development and the body response.

Besides, for other non-neoplastic disease, Braga et al. analyzed changes of ligand-receptor interactions in the airways of healthy individuals and patients with asthma through 10 × genomics. Unbiased analysis of cell–cell interactions identified that the cellular communication network dominated by mesenchymal-epithelial interactions in healthy airways, shifts to type 2 helper T cells (Th2) dominated interactome in asthmatic lung [61]. Using scRNA-seq to analyze ligand-receptor interactions between cells in diseased tissues can provide insight into the occurrence and progression of the disease.

### Pathogenic infection

Identification of pathogen targeted receptors and targeted cells is the key to treatment of pathogenic infections. Severe Acute Respiratory Syndrome Coronavirus 2 (SARS-CoV-2) has been declared a global public health emergency. Angiotensin I converting enzyme 2 (ACE2) is the host receptor by SARS-CoV-2 to infect human cells. To investigate whether there are other co-receptors/auxiliary proteins as ACE2 partner to facilitate virus entry, Furong Qi et al. explored the single cell gene expression atlas including 119 cell types of 13 human tissues which was mostly sequenced by 10 × genomics, and analyzed the single cell co-expression spectrum of 51 reported RNA virus receptors and 400 other membrane proteins. The result showed that the candidate co-receptors, manifesting the most similar expression patterns with ACE2 across 13 human tissues, are all peptidases, including ANPEP, DPP4 and ENPEP. The crosstalk between coronavirus targets and their surrounding cells indicated that macrophages frequently communicate with the coronavirus targets through chemokine and phagocytosis signaling which highlighting the importance of tissue macrophages in immune defense and immune pathogenesis [62]. Additionally, Qi-Lin Chen et.al. thought that cell receptor-related genes of SARS-CoV-2 is critical for understanding the pathogenesis of SARS-CoV-2 in various tissues, especially in the kidney. Their results showed that ACE2 was widely expressed in specific cell subgroups of various human tissues using 10X genomic, especially in intestinal epithelial cells, kidney proximal tubule (PT) cells, and also alveolar-type (AT) 2 cells of the lung [63]. These results indicate multiple routes for SARS-CoV-2 to infect with human cells/organs and suggest alternative strategies for therapeutic intervention. Studies of ligand-receptor interactions using scRNA-seq provided valuable reference data for the prevention and treatment of current SARS-CoV-2 infection, which are foundation for multi-organ multicellular therapy of pathogen infection.

### Physiological process

The cells of a multicellular organism are derived from a single zygote and genetically identical. Yet, they are phenotypically very different. This difference is the result of a process commonly called cell differentiation [64]. The essence of cell differentiation is the selective expression of intracellular genomes in time and space as cells are stimulated by external signals. Studying the signaling molecules communicated between cells during development is a good way to understand the mechanism of selective cell differentiation during the growth and development of the organism. Roser Vento-Tormo et.al. profiled the transcriptomes and cell–cell communication of about 70,000 single cells using Smart-seq2 and 10 × genomics technology, from first-trimester placentas with matched maternal blood and decidual cells. The results revealed the cellular organization of the decidua and placenta, and the interactions that are critical for placentation and reproductive success [65]. Additionally, Popescu et al. investigated the interaction between erythrocytes and macrophages by Smart-seq2 and 10 × genomics technology. The result showed that some important ligand&receptor such as VCAM1, ITGB1 and ITGA4, related to hematopoiesis in the fetal hematopoietic system [66]. During the development of the organism, immune cells play an important role in the development of the hematopoietic system.

### Pharmacological research

Moverover, the advent of various targeted drugs which are developed based on ligand-receptor interactions has solved many clinically difficult diseases and improved the survival of patients. However, targeted drugs have a narrow range of applicability, and many patients are unable to apply the targeted drugs or have poor therapeutic outcomes. Most current cancer patients do not respond positively to immune checkpoint blockers or have to discontinue their use due to significant side effects. for example, the positive percentage of patients for drugs related to targeting PD-1-PD-L1 rarely exceeds 40% [67].

Recently, Kathryn E Yost et.al. performed paired single-cell RNA and T cell receptor sequencing (10X droplet-based sequencing) on 79,046 cells from site-matched tumors from patients with basal or squamous cell carcinoma before and after anti-PD-1 therapy [68]. An increased frequency of follicular helper T cells (Tfh) cells and exhausted/activated CD8 + T cells appeared after anti-PD-1 treatment, which supporting that PD-1 blockade primarily impacts CD8 + T cells. Single cell T cell receptor sequencing (scTCR-seq) analysis indicated clonal replacement of exhausted clones when comparing pre- to post-treatment samples, suggesting that T cell receptor (TCR) dynamics of exhausted cells were mainly influenced by PD-1 blockade, not tumor biopsy timing or location. This may suggest that the effectiveness of targeted drug therapy is closely related to immune cell status. Combination of targeted drugs and immune cell therapy may enhance the therapeutic effect and improve patient survival.

Analysis of ligand-receptor interactions at the single-cell level has shown an important role in science research, helping researchers explore the mechanisms operating on immune cells in the microenvironment in depth. And there is expected to advance disease research and treatment.

## Analytical tools for ligand-receptor interactions at single-cell level

Now, multiple analysis tools for investigating cellular communications through ligand-receptor interactions were developed for deeper analysis of cell crosstalk based on scRNA sequencing (Table 2).

**Table 2:** The analytical tools for ligand-receptor interactions at single cell level

Tools	Type	Algorithm analysis rationale	Databases	Ligand-receptor complexs	Application	Author
General analysis						
ProximID [69]	Software	Expression level	No	No	Build a cellular network based on physical cell interaction and single-cell mRNA sequencing, discover new preferential cellular interactions without prior knowledge of component cell types	Jean-Charles Boisset et al.
iTALK [70]	R package	Expression level	No	No	Characterize and illustrate intercellular communication signals in the multicellular tumor ecosystem using single-cell RNA sequencing data	Yuanxin Wang et al.
PyMINEr [81]	Python package	Expression level	No	No	Detection of autocrine-paracrine signaling networks	Scott R. Tyler et al.
scTensor [82]	R package	Tensor decomposition	Yes	No	Detect some hypergraphs includingparacrine/autocrine cell–cell interactions patterns, which cannot be detected by previous methods	Koki Tsuyuzaki et al.
SoptSC [83]	R package	Cell–cell similarity matrix	Yes	No	Predict cell–cell communication networks, enabling reconstruction of complex cell lineages that include feedback or feedforward interactions	Shuxiong Wang et al.
cellTalker [71]	R package	Differentially expressed genes	No	No	Evaluate cell–cell communication	Anthony R Cillo et al.
CellPhoneDB [46]	Python package	Expression level	Yes	Yes	Predict enriched cellular interactions between two cell types from single-cell transcriptomics data	Mirjana Efremova et al.
SingleCellSignalR [45]	R package	Expression level	Yes	No	Provide a unique network view integrating all the intercellular interactions, and a function relating receptors to expressed intracellular pathways	Simon Cabello-Aguilar et al.
Signal pathways						
NicheNet [75]	R package	Weighting network	No	No	Infering ligands and their gene regulatory effects	Robin Browaeys et al.
CellChat [77]	R package	Weighting network	Yes	Yes	Predict major signaling inputs and outputs for cells and how those cells and signals coordinate for functions using network analysis and pattern recognition approaches	Suoqin Jin et al.
Spatical cellular communication						
SpaOTsc [79]	Python package	Spatial cell–cell distance and average enrichment of genes	No	No	(1) infer space-constrained cell–cell communications, (2) infer spatial distance for intercellular signaling, and (3) construct a spatial map of intercellular gene–gene regulatory information flow	Zixuan Cang et al.
CSOmap [78]	Matlab package	Abundance of interacting ligands and receptors, and their affinity	No	No	Reconstruction of cell spatial organization from single-cell RNA sequencing data based on ligand-receptor mediated self-assembly	Xianwen Ren et al.
Sequencing						
PIC-seq [80]	Sequencing technology	Sequencing physically interacting cells	No	No	Map in situ cellular interactions and characterizes their molecular crosstalk	Amir Giladi et al.

Databases: if there are databases constructed for these tools

Ligand-receptor complexs: if the structures of ligand-receptor comlpexs were considered by the tools

### General analysis

There are many analysis tools designed for cellular communications analysis through ligand-receptor interactions using scRNA-seq. ProximID [69] is an early algorithm for building a cellular network based on physical cell interaction and single-cell mRNA sequencing. It can be used to discover new preferential cellular interactions without prior knowledge of component cell types. And a Tac1 + enteroendocrine cell-Lgr5 + stem cell interaction in small intestine crypts was identified by ProximID. ProximID is a potent tool for the discovery of new prospective niches, especially when cell types and relative spatial positions are unknown. There's also iTALK [70] (https://github.com/Coolgenome/iTALK). This tool allows to customizate ligand-receptor libraries, and the analysis method is relatively simple and convenient. However, the default analysis species of this tool is human. If you want to analyze other species, there is a need to convert gene to the human corresponding gene.

CellTalker is widely used which was developed by Anthony et al. in 2020 January [71] (https://arc85.github.io/celltalker). This R package uses a recently described list of receptors and ligands (including soluble ligands such as cytokines) [40] to identify putative ligand-receptor interactions between cell types. And the algorithm is based on differential gene analysis. The ligands/receptors expressions of human papillomavirus (HPV) ± CD4 + T follicular helper cells that is associated with longer progression-free survival in HNSCC patients were significantly different found by cellTalker [71]. Additionally, in 2020 Apr, Teichmann laboratory and Vento-Tormo laboratory co-developed CellPhoneDB [46], which is a python package that's widely used currently. The main advantage of CellPhoneDB over other tools that takes into account the subunit architecture of both ligands and receptors, representing heteromeric complexes accurately. The researchers structured a novel repository of ligands, receptors and their interactions including heteromeric complexes which stores 1086 proteins, 501 are secreted proteins, 585 species as membrane proteins (Table 1). Eric Song et.al. found that in the cerebrospinal fluid (CSF) of COVID-19 patients, these activated innate immune cell populations are predicted to interact with CD8 and CD4 T cells applicating CellPhoneDB, suggesting a coordinated anti-viral immunological response occurring in the CSF of COVID-19 patients [72].

SingleCellSignalR is a new R package from Simon et al. in June 2020 [45], which used a new regularized product score to assess the confidence in predicted ligand-receptor interactions. It relies on a comprehensive database of known ligand-receptor interactions, which called LRdb (Table 1). LRdb compiled the content of existing databases and integrated informations of Reactome pathways [73] and Gene Ontology Cellular Compartment (GOCC) annotation. Therefore, singleCellSignalR has the abilities to represent a complete intercellular network and to import the latter in systems biology tools such as Cytoscape, and to explore receptor downstream signaling by integrating Reactome and Kyoto Encyclopedia of Genes and Genomes (KEGG) pathways. By mapping mouse genes to their human orthologs according to Ensembl [74] to exploit LRdb, the result of applicating SingleCellSignalR on mouse epidermis data discovered an oriented communication structure from external to basal layers.

### Signal pathways

In addition to these tools that only anlysis ligand-receptor interactons, there are tools which not only anlysis ligand-receptor interactons, but also explore downstream signal pathways. More recently, Yvan Saeys’s team proposed NicheNet [75] (https://github.com/saeyslab/nichenetr), which takes into account the changes in the downstream signaling network in the receiver cells, using the estimated downstream signaling changes to predict the activity of the upstream receptors, to identify the functionally influential cell–cell communication. The algorithm is based on a weighting network, which refines the intensity of the interaction and allows infer active ligands and their gene regulatory effects on interacting cells. Andrew L Ji et.al. found a tumor-specific keratinocyte (TSK) population unique to cancer, which localized to a fibrovascular niche. And TSK cells were a hub for intercellular communication revealed by NicheNet [76]. Recently, Suoqin Jin et.al. constructed a database of interactions among ligands, receptors and their cofactors that accurately represents known heteromeric molecular complexes. Based on mass action models, they then developed CellChat, a tool that is able to quantitively infer and analyze intercellular communication networks from scRNA-seq data [77]. Application of CellChat to several scRNA-seq datasets of mouse skin embryonic development and adult wound healing has demonstrated the ability to extract complex signal patterns, including previously known and new ones.

### Spatical cellular communications

Furthermore, Some researcheres believed that cellular communication analysis based on known ligand–receptor interactions could reshape tissue cell structure. CSOmap [78], SpaOTsc [79] are tools that attempt to reconstruct the spatial information of cells through ligand-receptor interactions. CSOmap successfully recapitulate the spatial organization of multiple organs of human and mouse including tumor microenvironments for multiple cancers in pseudo-space, and reveal molecular determinants of cellular interactions [78]. Differently, SpaOTsc not only tries to construct a spatial metric for cells in scRNA-seq data, but also reconstruct cell–cell communication networks by identifying intercellular regulatory relationships between genes.

### Sequencing

In addition to the aforementioned tools for intercellular interaction. do Amit and Amos Tanay jointly presented physically interacting cells sequencing (PIC-seq), which combines cell sorting of physically interacting cells (PICs) with single-cell RNA-sequencing [80]. PIC-seq systematically maps in situ cellular interactions and characterizes their ligand-receptor crosstalk by using computational modeling. Analysis of T cell- dendritic cells pairs reveals an interaction-specific program between pathogen-presenting migratory DCs and T cells. This method provides a direct and broadly applicable technology to characterize intercellular communication-specific pathways.

For general analysis, expression values of ligand receptor genes are used by many analytical tools to calculate the interaction, unlike cellTalker [71], which uses the differential expression of ligand receptor genes to perform the analysis. SoptSC [83] present similarity matrix-based optimization for single-cell data analysis. And the cell–cell relationships learned via the similarity matrix define which cells are clustered. The tools that include signal pathways analysis all use gene weighting networks with different algorithmic scoring points [75, 77]. The two analysis tools that incorporate the spatial localization of cells into the interaction factors differ significantly in their analysis algorithms. SpaOTsc [79] rely on structured optimal transport to recover spatial properties of scRNA-seq data by utilizing spatial measurements of a relatively small number of genes. SpaOTsc has broader applications, both in integrating non-spatial single-cell measurements with spatial data, and directly in spatial single-cell transcriptomics data to reconstruct spatial cellular dynamics in tissues. For CSOmap [78], the algorithmic process is composed of two main steps. The first is to estimate the cellular interacting potentials by integrating thousands of ligand-receptor pairs, resulting in a cell-by-cell affinity matrix. The second is to embed the inherently high-dimensional affinity matrix into three-dimensional space.

For these four classes of analysis tools, general analysis and signal pathways are more widely applicable and almost all scRNA-seq datas can be used for analysis. Spatical cellular communication analysis is suitable for analyzing tissue scRNA-seq datas, and the technology is not fully mature yet and needs to be combined with the results of wet experiments. Sequencing method which refer to PIC-seq, is suitable for dissecting cellular crosstalk of physically interacting cells, and could characterize intercellular interaction-specific pathways at high resolution. PIC-seq is a direct and broadly applicable technology [80].

The tools for ligand-receptor interaction at single cell level are collated in Table 2. These analytical tools and methods greatly facilitate researchers in quantifying the statistical significance of cell–cell interactions and reveal the potentially critical ligand-receptor pairs mediating such interactions. It will gain new insights into the role of cells.

## Inadequacy of current research

Although many tools have been developed and applied extensively in the study of receptors and ligands, there are still limitations of these tools. One problem with all the tools is the reliance on databases of known ligand-receptor pairs interactions. However, continuous update of current ligand-receptor libraries is necessary to cover more information of receptors, ligands and their interactions. Recently, receptor study of immunoglobulin superfamily (IGSFF) identified more than 60 new pairs of ligand -receptor [84]. The ligand-receptor pairs network is not completely understood and still needs futher exploration and improvement. Secondly, there is still a lack of understanding of ligand-receptor binding complexes at protein level, which the actual interactions occur, since most of the current studies of cellular interactions focus on genetic analysis. Post-transcriptional modification, binding mode, and the affinity strength etc. are yet to be thoroughly learnt. The integrated data which integrated the transcriptome profile with highly multiplexed proteomic and genomic data, was more informative than transcriptome data alone [85]. Thirdly, spatial location of tissue cells is vital. Spatiotemporal alterations in the microenvironment have a considerable impact on cells interactions. However, current single-cell sequencing technology cannot survey the specific spatial locations of cells. Lastly, the real cellular communication is carried out with single cell as a unit, which current researches are analyzed with cell types.

With the recognition of the complexity of the disease, personalized precision treatment is the core of treatment. Study of ligand-receptor pairs interactions using single-cell sequencing technology unveils the complex cell communication networks. Cellular communication is a very complicated process which is achieve through ligand-receptor signaling, and other mechanisms including pressure stimulation, concentration regulation, and intracellular signal transduction. Further extensive study is needed to get a deeper insight into these inter- and intra-cellular interactions for better understanding of disease progression and discovery of potential drug targets.

## Conclusion

Cell–cell communication governs the biological behaviors of multicellular populations. Ligand-receptor interactions, which is a vital type of cellular communication, have presented important roles in pharmacological research and disease progression as reviewed. Now, the emergence of scRNA sequencing technology gives a new way that are closer to the actual action of organisms for the research of ligand-receptor interaction. Many studies have learning cellular communication based on ligand-receptor interaction by scRNA sequencing (Fig. 2), to identify ligand-receptor interactions as biomarkers or potential therapeutic targets. Thus, tools or methods for learning ligand-receptor interactions by scRNA sequencing were developed, and sorted out in this study. It is expectant that us these tools to study diseases in more depth.

## Data Availability

Not applicable.

## Abbreviations

scRNA-seq: Single-cell RNA sequencing
FH: Familial Hypercholesterolemia
LDLR: Low density lipoprotein receptor
CTLA-4: Cytotoxic T lymphocyte-associated antigen-4
PD-1: Programmed death 1
ICOS: Inducible co-stimulator
TIM-3: T cell immunoglobulin and mucin domain 3
DLRP: Ligand-receptor pairing database
IUPHAR: International Union of Basic and Clinical Pharmacology
HPMR: Human plasma membrane receptor group
HPRD: Human Protein Reference Database
UniHI: Unified Human Interactome database
CellTalkDB: CellTalk database
MOAD: Mother of All Databases
GPCRs: G-protein coupled receptors
GLIDA: G protein coupled ligand-receptor pairs database
UCSF: University of California
CSCs: Cancer stem-like cells
LUAD: Lung adenocarcinoma
SARS-CoV-2: Severe Acute Respiratory Syndrome Coronavirus 2
ACE2: Angiotensin I converting enzyme 2
PT: Proximal tubule cells
Tfh: Follicular helper T cells
scTCR-seq: Single cell T cell receptor sequencing
TCR: T cell receptor
CSF: Cerebrospinal fluid
GOCC: Gene Ontology Cellular Compartment
KEGG: Kyoto Encyclopedia of Genes and Genomes
TSK: Tumor-specific keratinocyte
PIC-seq: Physically interacting cells sequencing
PICs: Physically interacting cells
IGSFF: Immunoglobulin superfamily
